# Significance of pulmonary resection in patients with metachronous pulmonary metastasis from pancreatic ductal adenocarcinoma: a retrospective cohort study

**DOI:** 10.1186/s12893-021-01236-w

**Published:** 2021-05-05

**Authors:** Taro Mashiko, Akira Nakano, Yoshihito Masuoka, Seiichiro Yamamoto, Soji Ozawa, Toshio Nakagohri

**Affiliations:** Department of Gastroenterological Surgery, Tokai University School of Medicine, 143 Shimokasuya, Isehara, Kanagawa 259-1193 Japan

**Keywords:** Pancreatic ductal adenocarcinoma, Metachronous pulmonary metastasis, Pulmonary resection, Metastasectomy

## Abstract

**Background:**

Pulmonary metastases from pancreatic ductal adenocarcinoma (PDAC) are relatively rare. Systemic chemotherapy is the first choice of treatment in patients with distant metastases, and the role of metastasectomy is controversial. The aim of the present study was to evaluate the outcome of patients with pulmonary metastases after resection of PDAC and the indications for metastasectomy.

**Methods:**

We retrospectively analysed patients with pulmonary metastases as the first recurrence after resection of primary PDAC between January 2006 and December 2018. Clinical data were obtained from the patients’ medical records. Relapse-free survival (RFS) and overall survival (OS) were analysed using the Kaplan–Meier method, and statistical significance was evaluated by the log-rank test.

**Results:**

Of the 417 patients with resected PDACs, 24 (7.9%) had pulmonary metastases. Six patients (25.0%) underwent pulmonary resection and 18 (75.0%) received systemic chemotherapy and best supportive care. There were no major complications requiring therapeutic intervention after pulmonary resection. The median RFS was 24.0 months (95% CI 10.8–37.2), and the 1-, 3-, and 5-year RFS rates were 66.7%, 33.3%, and 4.2%, respectively. The median OS was 50.0 months (95% CI 15.9–84.1), and the 1-, 3-, and 5-year OS rates were 95.8%, 70.3%, and 46.4%, respectively. All patients with resected pulmonary metastases were alive at the end of the study, whereas the median OS of the patients who did not undergo resection was 37.0 months (95% CI 34.4–39.6). Therefore, patients with resected pulmonary metastases had a significantly better prognosis (p = 0.008).

**Conclusions:**

Pulmonary resection may improve the prognosis in selected patients with pulmonary metastases from PDAC. However, the present study is based on a small number of patients and may include a selection bias; therefore, a multi-institutional prospective study is needed to clarify the indications for pulmonary resection.

## Background

Despite the development of multidisciplinary treatment for pancreatic ductal adenocarcinoma (PDAC), the prognosis for PDAC is still poor and the 5-year survival rate is less than 20% because of the high rate of recurrence [1, 2]. Even the most recent clinical trials of chemotherapy for distant metastases from PDAC have indicated a poor prognosis, with the median overall survival (OS) ranging from 8.5 months to 11.1 months [3, 4]. Recurrent PDAC often spreads to multiple organs. The most common site of PDAC metastasis is the liver, and the prognosis of liver-metastatic PDAC is extremely poor [5]. Conversely, the incidence of metachronous pulmonary metastasis from PDAC is relatively low, at 2.9–21.8% [6–8]. Metachronous pulmonary metastases of PDAC are also reported to have a better prognosis than recurrent PDACs in other sites [9–11]. A few reports have suggested the feasibility and efficacy of surgical resection for isolated pulmonary metastases of PDAC [12–15]. However, the role of surgical resection of recurrent PDAC remains controversial. The aim of the present study was to evaluate the outcome of patients with pulmonary metastases after resection of PDAC and the indications for metastasectomy.

## Methods

### Patients

We retrospectively analysed patients with pulmonary metastases as the first recurrence after surgery for primary PDAC between January 2006 and December 2018 in the Tokai University Hospital, Japan. Staging of the primary PDAC was performed using the Union for International Cancer Control tumour-node-metastasis (TNM) classification, 8th edition. We excluded patients with synchronous distant metastases, those who had undergone conversion surgery and those with pancreatic pathologies other than PDAC, such as intraductal papillary mucinous carcinoma, adenosquamous carcinoma, carcinosarcoma, and anaplastic carcinoma.

### Follow-up

All patients received routine postoperative surveillance. Patients receiving postoperative adjuvant chemotherapy underwent tumour marker measurement (carcinoembryonic antigen and colorectal carcinoma antigen) monthly for the first 6 months, then every 3 months until 3 years after surgery and every 6 months thereafter. Patients not receiving postoperative adjuvant chemotherapy underwent tumour marker measurement every 3 months. Chest and abdominal computed tomography was performed every 3 months for the first 3 years, every 6 months for the following 2 years and annually thereafter.

### Histological assessment and immunohistochemistry of pulmonary metastasis of PDAC

In patients who underwent pulmonary resection, the metastatic lesion was differentiated from primary lung cancer by immunohistochemistry, and the final diagnosis was confirmed in resected specimens pathologically using cytokeratin (CK) 7, CK20, thyroid transcription factor-1 (TTF-1), and napsin A. Pulmonary metastasis of PDAC has been reported to be CK7- and CK20-positive, but TTF-1- and napsin A-negative by immunostaining [16–18].

### Statistical analysis

All of the statistical analyses were performed using a standard statistical program (SPSS software for windows, version 26.0; Chicago, IL, USA). Chi-square and Mann–Whitney U tests were used to analyse patient parameters. OS and recurrence-free survival (RFS) were analysed using the Kaplan–Meier method, and statistical significance was evaluated by the log-rank test. A p-value less than 0.05 was considered statistically significant.

## Results

### Baseline characteristics of patients with pulmonary metastasis of PDAC

Of the 417 patients with resected PDACs, 304 (72.9%) experienced recurrence during the observation period. Only 24 (7.9%) patients had pulmonary metastases as the first recurrence. The median observation period was 44 months (10–153 months). Baseline characteristics of these 24 patients at the time of surgical resection of the primary PDAC are shown in Table 1. Fifteen men and 9 women were included in our study, with a median age of 69 years (55–80 years). Gemcitabine combined with S-1 therapy was administered to two patients as neoadjuvant chemotherapy (8.3%). For resection of the primary tumour, patients underwent pancreaticoduodenectomy (n = 17; 70.8%) or distal pancreatectomy (n = 7; 29.2%). Six patients (25.0%) underwent portal vein resection and reconstruction. Five, 1, 8, and 10 patients had TNM stage 1A, 1B, 2B, and 3 disease, respectively. For adjuvant chemotherapy, patients received gemcitabine (n = 4), gemcitabine combined with S-1 (n = 3) or S-1 (n = 14); three patients opted not to receive adjuvant chemotherapy. If recurrence was detected during routine follow-up, the regimen was changed. After the diagnosis of pulmonary metastasis, 6 patients (25.0%) underwent pulmonary resection and 18 (75.0%) received chemotherapy or best supportive care. Best supportive care was provided to three patients with poor performance status and one patient with renal dysfunction who could not receive chemotherapy. The median diameter of the largest tumour in the pulmonary metastases was 11.5 mm (3–32 mm).

**Table 1:** Baseline characteristics of patients with metachronous pulmonary metastases from PDAC

Variables	
Age, years, median (range)	69.0 (55.0–80.0)
Sex, n (%)	
Male	15 (56.0)
Female	9 (44.0)
ECOG performance status, n (%)	
0,1	21 (87.5)
2,3	3 (12.5)
Location, n (%)	
Head	17 (70.8)
Body-tail	7 (29.2)
CEA (ng/mL), median (range)	3.9 (1.2–51.5)
CA19-9 (U/mL), median (range)	128.5 (11.0–1185.9)
Neoadjuvant chemotherapy, n (%)	
Gemcitabine + S-1	2 (8.3)
Surgical procedure, n (%)	
PD	17 (70.8)
DP	7 (29.2)
Portal vein resection, n (%)	6 (25.0)
Primary tumour size (mm), median (range)	31 (6–80)
Pathologic T category (primary tumour), n (%)	
T1	6 (25.0)
T2	11 (45.8)
T3	7 (29.2)
Pathologic N category, n (%)	
N0	6 (25.0)
N1	8 (33.3)
N2	10 (41.7)
Residual tumour category, n (%)	
R0	21 (87.5)
R1	3 (12.5)
Tumour differentiation, n (%)	
Well	9 (37.5)
Moderate	11 (45.8)
Poor	4 (16.7)
Pathologic UICC stage, n (%)	
1A	5 (20.8)
1B	1 (4.2)
2A	0
2B	8 (33.3)
3	10 (41.7)
Adjuvant chemotherapy, n (%)	21 (87.5)
S-1	14 (58.3)
Gemcitabine	4 (16.7)
Gemcitabine + S-1	3 (12.5)

*PDAC* pancreatic ductal adenocarcinoma, *PD* pancreatoduodenectomy, *DP* distal pancreatectomy, *ECOG* Eastern Cooperative Oncology Group, *UICC* Union for International Cancer Control, *CEA* carcinoembryonic antigen, *CA19-9* colorectal cancer antigen

A single metastasis was observed in 1 case, 2–5 metastases in 11 cases, 6–9 metastases in four cases, 10–19 metastases in 3 cases, and more than 20 metastases in five cases. Of the six pneumonectomies, two were biopsied preoperatively to confirm the diagnosis of pulmonary metastases from PDAC. The other four cases were not clearly differentiated from primary lung cancer and were determined only by computed tomography. In our department, the indication for resection of pulmonary metastases is good performance status and localised and curative resection regardless of single or multiple metastases. On the other hand, patients in a poor general condition, patients with early recurrence (within 1 year of surgery), and patients with multiple metastases that were not localised were excluded from surgery.

### RFS and OS of all patients

**Fig. 1:**
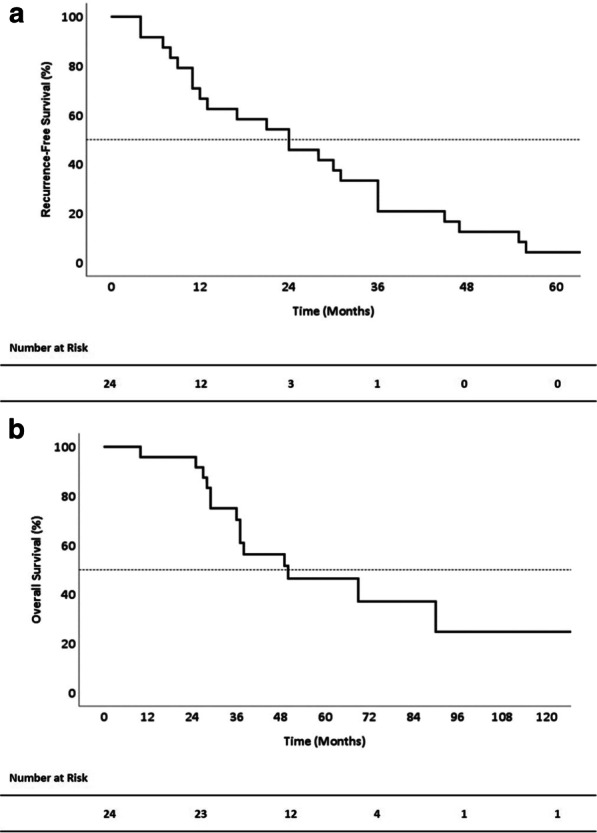
Survival of patients with pulmonary metastases after resection of primary PDAC. **a** Recurrence-free survival and **b** Overall survival. *PDAC* pancreatic adenocarcinoma

The median RFS of all 24 patients was 24.0 months (10.8–37.2 months), and the 1-, 3-, and 5-year RFS rates were 66.7%, 33.3% and 4.2%, respectively (Fig. 1a). The median OS was 50.0 months (15.9–84.1 months), and the 1-, 3-, and 5-year OS rates were 95.8%, 70.3%, and 46.4%, respectively (Fig. 1b).

### Treatment of patients with pulmonary metastasis of PDAC

Table 2 shows the clinical courses of the six patients who underwent pulmonary resection. No lobectomies were performed, and all patients underwent video-assisted thoracoscopic partial pulmonary resection. At the time of the first operation, resection of a single nodule was performed in four patients, whereas two patients underwent resection of two or more lesions. Three patients underwent two or more pulmonary resections. In all cases of resection of pulmonary metastases, the resection margins were negative. The median tumour diameter was 13 mm (3–31 mm). CK-7, CK20, and TTF-1 assays were performed for all patients. Napsin A and CA19-9 immunostainings were performed in two and three patients, respectively. The metastases were finally diagnosed by two pathologists as consistent with pulmonary metastases of PDAC. In this study, no lung lesions were diagnosed as primary lung cancer.

**Table 2:** Clinical course of patients with pulmonary metastases from PDAC who received pulmonary resection

Case	Age/Sex	PDAC surgery	pStage	Adjuvant chemotherapy	RFS (Months)	PM location (tumour number)	Neoadjuvant chemotherapy	PM surgery	2nd adjuvant chemotherapy	RFS (PM surgery) (months)	2nd recurrence site	2nd recurrence treatment	OS (Months)
1	72M	PPPD + PVR	2B	-	36	Left upper lobe (1) Left lower lobe (1)	–	Partial resection	–	98	–	–	132 (Alive)
2	66F	SSPPD	3	S-1	47	Right upper lobe (1) Right lower lobe (2)	–	Partial resection	–	13	–	–	60 (Alive)
3	68M	SSPPD + PVR	3	S-1	24	Right upper lobe (1) Left upper lobe (1)	–	Partial resection	–	3	Right pulmonary metastases	GEM + nab-PTX → FFX	55 (Alive)
4	70M	SSPPD + PVR	2B	S-1	13	Left lower lobe (2)	–	Partial resection	–	3	Right pulmonary metastases	GEM + nab-PTX	32 (Alive)
5	71M	DP	3	S-1	31	Right upper lobe (1) Right Lower lobe (1)	GEM + nab-PTX	Partial resection	GEM + nab-PTX → GEM	9	–	–	53 (Alive)
6	80M	DP	1A	S-1	36	Right upper lobe (1) Left upper lobe (1)	GEM + nab-PTX	Partial resection	–	3	–	–	59 (Alive)

*PPPD* pylorus-preserving pancreaticoduodenectomy, *SSPPD* subtotal stomach preserving pancreaticoduodenectomy, *DP* distal pancreatectomy, *PVR* portal vein resection, *PM* pulmonary metastasis, *GEM* gemcitabine, *nab-PTX* nab-paclitaxel, *FFX* FOLFIRINOX, *RFS* recurrence-free survival

Table 3 compares the clinicopathological features of patients who did and did not undergo pulmonary resection. Patients who underwent pulmonary resection were significantly more likely to have unilaterally localised disease (p = 0.006), but there was no significant difference between the two groups in any other clinicopathological features.

**Table 3:** Baseline characteristics of the patients who did or did not receive pulmonary resection

Variables	Pulmonary resection (n = 6)	No pulmonary resection (n = 18)	p-value
Age, years, median (range)	70.5 (66.0–80.0)	70 (55.0–80.0)	0.251
Sex (Male/Female)	5/1	10/8	0.238
ECOG Performance status (0,1/2,3)	6/0	21/3	0.403
CEA (ng/mL), median (range)	4.9 (1.5–51.5)	3.7 (1.2–13.0)	0.251
CA19-9 (U/mL), median (range)	249.3 (18.0–955.1)	90.1 (11–1185.9)	0.224
Primary tumour location (Head/Body-tail)	4/2	11/7	0.603
Primary tumour size, median (range)	32.5 (20.0–80)	31 (6.0–50.0)	> 0.99
Pathologic T category of primary tumour (T1,2/T3)	4/2	13/5	0.586
Pathologic N category of primary tumour (N0,1/N2)	4/2	11/7	0.603
Pathologic UICC Stage (1,2/3)	3/3	11/7	0.494
Residual tumour category (R0/R1)	6/0	15/3	0.403
Tumour differentiation (Well/Moderate, poor)	2/4	7/11	0.603
Completion of adjuvant chemotherapy, n (%)	3/3	5/13	0.302
Tumour diameter of pulmonary metastases (mm), median (range)	13.0 (3.0–31.0)	11.5 (4.0–32.0)	0.923
Number of PM (solitary/multiple)	0/6	1/17	0.750
Site of PM (unilateral/bilateral)	4/2	1/17	0.006

*CEA* carcinoembryonic antigen, *CA19-9* colorectal cancer antigen, *UICC* Union for International Cancer Control, *PM* pulmonary metastases, *ECOG* Eastern Cooperative Oncology Group

### Postoperative complications of pulmonary resection

Complications included pleural effusion in one patient. No patients had complications of Clavien–Dindo category 3 or higher, and no patients required transfusion. The median hospital stay was 9 days (7–11 days).

### Univariate analysis of prognostic factors

**Fig. 2:**
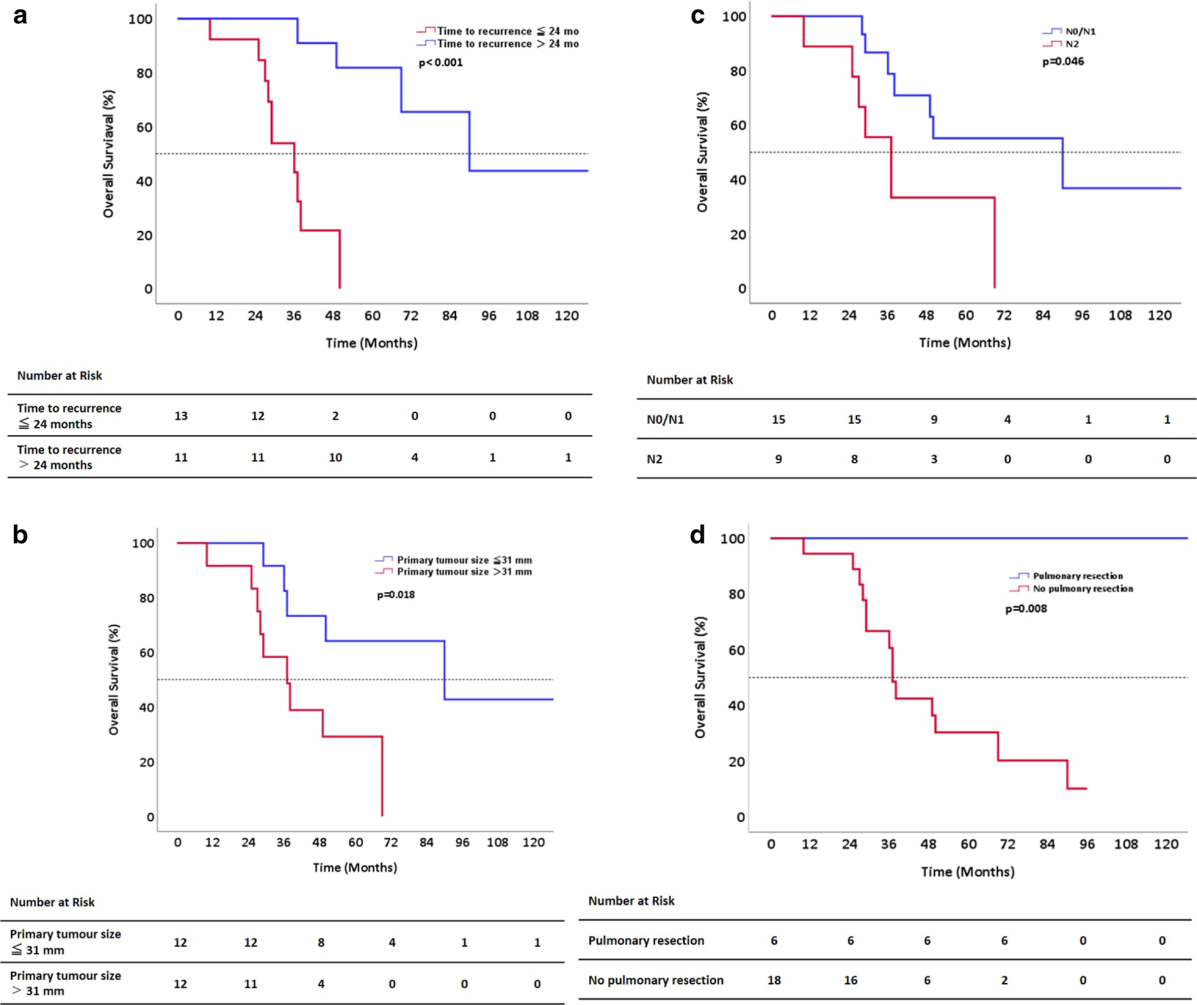
Univariate analysis of prognostic factors. **a** Kaplan–Meier curves of overall survival according to the time to development of pulmonary metastases after resection of the primary PDAC (≤ 24 months versus > 24 months). **b** Kaplan–Meier curves of overall survival according to primary tumour size (> 31 mm versus < 31 mm). **c** Kaplan–Meier curves of overall survival according to pathological N stage (pN0/N1 versus pN2). **d** Kaplan–Meier curves of overall survival according to pulmonary resection (pulmonary resection versus no pulmonary resection). *PDAC* pancreatic ductal adenocarcinoma

There was no significant difference in prognosis between patients with single and multiple pulmonary metastases (p = 0.523). The OS was 36 months (95% CI 27.2–44.8 months) and 90.0 months (95% CI 49.9–130.1 months), respectively, for patients who developed pulmonary metastases ≤ 24 months and ≥ 24 months after resection of the primary tumour (p < 0.001) (Fig. 2a). The OS was 90 months (95% CI 16.9–163.1) and 37 months (95% CI 23.6–50.4), respectively, for patients with primary tumours ≤ 31 mm and those with tumours > 31 mm (p = 0.018) (Fig. 2b). There was no difference in prognosis according to the presence or absence of lymph node metastasis (p = 0.213). However, when comparing patients with N2 disease with four or more regional lymph node metastases to patients with N0 and N1 disease, the OS was 37 months (95% CI 25.9–48.1) and 90 months (95% CI 15.0–165.0), respectively (p = 0.046) (Fig. 2c). The median RFS was 31 months (95% CI 21.4–40.6) and 17 months (95% CI 0.0–35.7), respectively, for patients who underwent pulmonary resection and those who did not. Although there was no significant difference in disease-free survival (DFS) in the small number of cases studied (p = 0.643), early recurrence within 1 year occurred more frequently in the no pulmonary resection group (eight cases). This group also had a shorter DFS. Conversely, all patients with resected pulmonary metastases survived, whereas the OS of patients who did not receive pulmonary resection was 37.0 months (95% CI 34.4–39.6), indicating a significantly better prognosis of patients who underwent pulmonary metastasectomy (p = 0.008) (Fig. 2d).

## Discussion

PDAC can be cured only by multidisciplinary treatment with surgery and postoperative adjuvant chemotherapy. However, the recurrence rate is high, with 80% of patients reporting a relapse within 2 years [5]. Additionally, PDAC often metastasises to multiple organs by hematogenous, lymphogenic, and perineural routes or by direct invasion [19, 20].

According to a meta-analysis including 83 studies, the liver is the most common site of metastasis of PDAC, accounting for 26.5% of recurrences, followed by local recurrence (20.8%), peritoneal dissemination (13.5%), and lung metastasis (11.4%) [21]. The mechanism underlying pulmonary metastasis as the first recurrence is not well understood, but it has been suggested that this occurs through lymphatic entry into the macrocirculation from lymph node metastasis or hematogenous metastasis via the collateral blood circulation around the spleen due to congestion of the splenic vein [22].

The time to recurrence has been reported to be longer in patients with pulmonary metastasis as the first recurrence than in patients with other sites of recurrence [9–11, 14, 15, 21, 22]. Zheng et al. noted that these patients are more likely to have a lower pathological T category and less vascular invasion than patients with metastases at other sites [22]. In our study, the median RFS and OS of patients with pulmonary metastases were 24 months and 50 months, respectively, similar to those in previous reports [7, 14, 22]. On the contrary, most of the cases in our study were T2 or larger with relatively large tumour diameters.

Resection of recurrent PDAC lesions is usually not indicated, and systemic chemotherapy is administered instead. However, in our study, the prognosis of patients with resected pulmonary metastases was significantly better than that of patients who did not undergo resection. Arnautakis et al. reported that nine patients who underwent pulmonary resection had a longer OS than 22 patients who did not (51 months versus 23 months, p = 0.04). They also demonstrated that pulmonary metastasectomy could be performed safely without major postoperative complications [7]. In our study, pulmonary resection was performed safely, and there were no complications requiring therapeutic intervention. Although all of our pneumonectomies were video-assisted thoracoscopic partial resections, we believe that pneumonectomy can be safely performed even after PDAC resection in patients who are in a good general condition. Thomford et al. advocated the following indications for the resection of pulmonary metastases: (1) tolerability of surgery, (2) controlled primary lesion, (3) no metastases in other organs, and (4) radiographic evidence of unilateral pulmonary metastasis [23].

Patients with poorly differentiated PDAC at the time of the initial surgery have a poor prognosis [24], and patients with pulmonary metastatic lesions less than 16 mm in size and stage 1 primary tumours have a better prognosis [25]. By contrast, Kruger et al. considered that patients with fewer than 10 localised metastases on one side of the lung had a good prognosis and that the size of each metastasis was not an important factor in planning pulmonary resection [10]. In our study, the prognosis was worse in patients with larger tumours and more than four metastatic lymph nodes in the primary PDAC. However, there was only one case of single metastasis. Therefore, it was difficult to determine if single or multiple metastases affected prognosis in this study. Thomas et al. reported a significantly better prognosis in patients with recurrence ≥ 21 months after PDAC resection than in those with recurrence ≤ 21 months after PDAC resection (92.3 months versus 31.3 months, p = 0.033). In addition, Ilmer et al. reported that the prognosis of patients with recurrence ≥ 17 months after PDAC resection was significantly more favourable than that of patients with recurrence ≤ 17 months after PDAC among patients undergoing pneumonectomy (32.2 months versus 14.8 months, p = 0.025) [24, 26]. Our study also showed a significantly better prognosis for patients who relapsed ≥ 24 months after PDAC resection. When examining patients who underwent pneumonectomy, two patients with recurrence ≤ 24 months after PDAC showed early recurrence after pneumonectomy. Therefore, the time to recurrence is an important factor when considering the indication for pneumonectomy.

Patients whose pulmonary metastases have been well-controlled by chemotherapy are also good candidates for surgery, but there is insufficient evidence on whether and when to perform pneumonectomy after chemotherapy. Two patients underwent resection after neoadjuvant chemotherapy for pulmonary metastases: one patient was treated with gemcitabine plus nab-paclitaxel for more than one year and was found to have no disease progression, and the other, who was older, was treated with gemcitabine plus nab-paclitaxel for one month before undergoing surgery at his request. Nakajima et al. also mentioned that adjuvant chemotherapy after curative resection of pulmonary metastasis from PDAC was controversial [25]. Therefore, only one of our patients received postoperative adjuvant chemotherapy after pneumonectomy, and the other five patients were only followed up. Despite the long duration of this study, there were no major changes in the techniques used or extent of lymph node dissection. Eleven patients received gemcitabine plus nab-paclitaxel and seven patients received other regimens. However, no bias was observed in the regimens between the two groups (p = 0.103), and thus, this did not affect the results of the study.

There are some limitations to this study. First, this is a retrospective study. Second, this study is based on a small number of cases and there may be selection bias. In addition, the no pulmonary resection group included cases of best supportive care. Due to the limited number of patients, we were unable to identify prognostic factors in patients with pulmonary metastases from PDAC using multivariate analysis. Further multicenter prospective studies are needed to determine the factors associated with prognosis in patients with pulmonary metastases from PDAC who undergo metastasectomy.

## Conclusions

Despite the limitations mentioned in the Discussion, our study showed that pulmonary resection may further improve prognosis in selected patients with pulmonary metastases from PDAC. In particular, patients with a longer time to recurrence for whom radial resections are suitable are considered to be good candidates for pulmonary resection. A multi-institutional prospective study is needed to clarify the indications for pulmonary resection. 

## Data Availability

The datasets generated and/or analysed during the current study are available from the corresponding author on reasonable request.

## Abbreviations

PDAC: Pancreatic ductal adenocarcinoma
CK: Cytokeratin
TTF-1: Thyroid transcription factor-1
RFS: Relapse-free survival
OS: Overall survival
DFS: Disease-free survival
TNM: Tumour-node-metastasis
CI: Confidence interval
